# GATA Binding Protein 3 as a Diagnostic Biomarker and Immune Infiltrates Regulator in Stomach Adenocarcinoma

**DOI:** 10.7759/cureus.91708

**Published:** 2025-09-06

**Authors:** Tasneem A Ahmed, Israa M Faris, Enas A Eisa, Shyma Musa, Amna Makawi, Mohamed Alfaki

**Affiliations:** 1 Zoology, Faculty of Science, University of Khartoum, Khartoum, SDN; 2 Immunology and Microbiology, Eldaein University, Eldaein, SDN; 3 Biochemistry, Al Neelain University, Khartoum, SDN; 4 Medicine and Surgery, University of Kordofan, El-Obeid, SDN; 5 College of Medicine, Elrazi University, Khartoum, SDN; 6 Computer Science, Al-Neelain University, Khartoum, SDN

**Keywords:** dna methylation, gata3, immune infiltration, pan-cancer analysis, stomach adenocarcinoma (stad)

## Abstract

Introduction

GATA3 is a transcription factor that regulates cell differentiation, proliferation, and immune function. While it is well recognized as a diagnostic marker in breast and urothelial cancers, but in stomach adenocarcinoma (STAD) remains unclear. This study aimed to investigate the expression, clinical significance, promoter methylation, immune infiltration, and genetic alterations of GATA3 in STAD using comprehensive bioinformatics approaches. To our knowledge, this is the first study to systematically evaluate GATA3 across multiple datasets in the context of gastric cancer.

Methods

The analyses were conducted on publicly available datasets, including Tumor Immune Estimation Resource (TIMER), Gene Expression Profiling Interactive Analysis (GEPIA), University of Alabama at Birmingham Cancer Data Analysis Portal (UALCAN), Kaplan-Meier plotter (KM Plotter), cBioPortal for Cancer Genomics, and Gene Expression Omnibus (GEO).

Results

Our results revealed that GATA3 is significantly upregulated across multiple cancer types, with the highest expression observed in STAD (p < 0.001). According to cancer stages I-IV, the expression increased in stages II-III but declined in stage IV, suggesting a role in tumor progression. However, survival analyses across several platforms consistently demonstrated no significant association between GATA3 expression and overall survival (p > 0.05). Immune infiltration analysis showed strong correlations between GATA3 expression and CD8+ T cells, CD4+ T cells, and macrophages (p < 0.0001), highlighting its potential involvement in shaping the tumor immune microenvironment. Interestingly, despite its upregulation, GATA3 exhibited elevated promoter methylation, suggesting additional regulatory mechanisms beyond epigenetics. Genetic alterations were rare (4%) and did not impact survival outcomes.

Conclusion

Collectively, these findings suggest that GATA3 is significantly upregulated in STAD and may serve as a novel diagnostic marker associated with immune infiltration. The study provides a foundation for future research exploring GATA3 as a potential target for diagnostic development and immunotherapy strategies in gastric cancer.

## Introduction

Stomach adenocarcinoma (STAD) is considered the third most lethal and the fifth most common malignancy globally [1]. Adenocarcinoma is a malignant neoplasm that develops from the epithelial cells of the glands or glandular structures and may arise in many parts of the body, including the breast, lung, prostate, gastrointestinal tract, containing the colon, rectum, pancreas, stomach, and esophagus [2]. STAD develops from the epithelial cells of the gastric glands. It can develop in any part of the stomach, including the cardia, body, antrum, and pylorus [3]. Consequently, STAD occurs distally or proximally, where distal stomach cancer is strongly associated with *Helicobacter pylori *infection and poor dietary intake of antioxidant vitamins such as A, C, and E; however, the microbiome of stomach still needs to be investigated more to draw clear conclusions of such an association even though there has been prior insight of engaging bacteria as non-invasive biomarker as they are clearly involved in stress-response and metastatic response to agitate the microflora of stomach [4-6].

Proximal stomach cancer is connected with gastroesophageal reflux disease (GERD), obesity, high fat intake, and medium to high socioeconomic status. Despite the STAD pathogenesis research advancement, therapeutic approaches have been gradually developed [7]; besides surgical intervention, radiotherapy, and chemotherapy, molecular targeted therapy has also appeared as a potential method, but more understanding is required of the sublevels of interactive chronic inflammatory response, immune suppressive modulation, and the carcinogenic effects which result in its efficient therapeutic approach [8-10]. With the increasing mortality rate due to STAD, it is necessary to understand that the low survival rate is due to late diagnosis, particularly at progressive stages; consequently, earlier detection of STAD is significant.

GATA is known as a transcription factor family that binds to specific DNA sequences through its zinc finger domains by sharing an amino acid sequence in their DNA-binding domain, and contains six members, sub-grouping into GATA1-3 and GATA4-6, which all play roles in gene expression, cell proliferation, immunosuppressive, or even tissue development. GATA binding protein 3 (GATA3) hasn’t been investigated well in association with cancers, including STAD [11,12]. GATA3 is more linked to the regulation of T cell differentiation, proliferation, and adhesion, and implicates both thymic progenitors and peripheral T cells [13]. Recent studies have highlighted GATA3's role in T-cell lymphoproliferative disorders, emphasizing its significant role in gene expression as it might behave either oncogenically or as a suppressor in relation to the health and disease microenvironment.

In this study, we aimed to investigate the expression of GATA3 in stomach adenocarcinoma and its potential use as a diagnostic, prognostic biomarker, and therapeutic target. We hypothesized that GATA3 expression levels are significantly associated with patient outcomes in stomach adenocarcinoma and that it may serve as a novel diagnostic and prognostic biomarker for this disease due to its gene expression and survival rate correlation.

## Materials and methods

Gene expression analysis

GATA3 gene expression was analyzed across different cancers using the Tumor Immune Estimation Resource (TIMER.2) database [14]. TIMER uses data obtained from The Cancer Genome Atlas (TCGA) [15], analyzed based on the Wilcoxon test to assess the statistical significance of differential expression patterns. It was employed to investigate the gene differential expression between normal and tumor tissues with a sample size of 10,897 tumors from 32 cancer types. 

We used the Gene Expression Profiling Interactive Analysis (GEPIA) database [16] to explore the gene expression further by utilizing the differential DIY, boxplot module to show the GATA3 expression across STAD, applying the criteria of p-value ≤ 0.05, and log2 fold change (log2FC) ≥ 1 to determine the statistical significance.

The University of Alabama at Birmingham Cancer Data Analysis Portal (UALCAN) [17] was used to confirm GATA3 expression outcomes from both GEPIA and TIMER databases, and examine the association between GATA3 expression and clinical pathological parameters of cancer, since UALCAN provides more critical genomic analyses covering a sample size of 1871 pre-miRNAs in 32 cancer types.

The Human Protein Atlas (HPA) [18] was used to examine the histological differences in STAD as it is considered a comprehensive human protein map covering various cells from brain, tissues, cell lines, or subcellular locations, and most significantly cancers, which contribute to understanding and validating the clinical pathological findings from UALCAN, based on the significant statistical differences for cancer stage, gender, race, and age.

Prognostic evaluation of GATA3 in STAD

To examine the *GATA3* gene in STAD as a prognostic marker, correlation of overall survival analysis and gene expression over the period from the patient’s diagnosis or treatment is used. A Kaplan-Meier plotter (KM Plotter) [19], GEPIA, and UALCAN were applied to the sample size of KM, using Cox proportional hazards regression as a statistical tool.

Gene-immune cell infiltration analysis

Another approach for using the TIMER2.0 platform was to utilize the analysis of immune infiltration of various cancer types systematically to understand their interaction between immune cells and gene expression. It is used to estimate the abundance of six immune cells, including B cells, CD4+ T cells, CD8+ T cells, neutrophils, macrophages, and dendritic cells. In our study, we primarily used it to investigate *GATA3* gene expression in STAD and its relationship with immune infiltration, which would influence any immune therapy resulting in the outcomes of survival rate. A scatter plot was created to illustrate the correlation based on Spearman's Rho value. Therefore, statistical significance for immune cell infiltration was determined using a p-value ≤ 0.05.

DNA methylation analysis

For studying the relationship between *GATA3* gene upregulation in STAD and DNA methylation, two different methylation tools were used to analyze the DNA promoter, including methylation analysis by UALCAN and DNA Methylation Interactive Visualization Database (DNMIVD) [20].

DNMIVD was built to integrate comprehensive genomic data provided by TCGA and Gene Expression Omnibus (GEO), which are based on throughput, sequenced, and annotated DNA methylation. Spearman correlation and Kaplan-Meier curve with p-value <0.05 were utilized to draw the relationship of DNA methylation with GATA3 upregulation.

Genomic analysis of gene alterations

Understanding the genomic nature of a gene gives more insights into gene expression, epigenetic defects, or the prognosis of a disease. The cBioPortal database [21] provides critical genomic analyses, including alterations, copy number segments, variants, correlations, survival rate, mutation types, etc. 

To identify the types of *GATA3* gene alterations and other mutation frequencies in association with STAD, we applied the TCGA Pan-Cancer Atlas Studies, which included 32 studies of 10,967 samples, to understand the genomic and molecular behavior of GATA3.

Lab-based validation

To validate the findings, we utilized datasets from the National Center for Biotechnology Information (NCBI), GEO [22], which is available publicly. The dataset ID GSE29272 has gene expressions for cardia and non-cardia gastric cancer samples. Therefore, GEO2R was employed for the differential gene expression analysis by comparing the tumor samples and normal samples. The results were subsequently visualized using SRplot to generate a volcano plot depicting gene expression differences with Log2FC >0.6 and adjusted p-value < 0.05 [23].

Statistical analysis

Transcriptomic values were log2(TPM+1) transformed. For GEPIA2 and UALCAN, two-tailed Student’s t-tests were used, and for TIMER2.0, the Wilcoxon test was used. Boxplots displayed group differences with significance of p < 0.05, p < 0.01, and p < 0.001, respectively. Purity-adjusted Spearman’s correlations were used for immune infiltration analyses. Kaplan-Meier analyses (GEPIA2, UALCAN, KM Plotter) were used to compare overall survival (OS) and disease-free survival (DFS) between expression groups; hazard ratios (HRs) and 95% CIs were reported. All analyses were cross-validated across multiple platforms to ensure robustness of the findings.

## Results

Gene expression analysis

**Figure 1:**
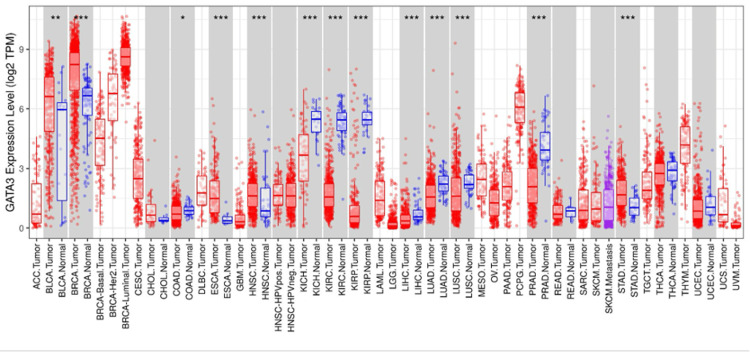
Pan-cancer analysis of GATA3 expression levels using TIMER in various cancers *p < 0.05, **p < 0.01, ***p < 0.001 was assumed to be significant. TIMER: Tumor Immune Estimation Resource [15]; BLCA: bladder urothelial carcinoma; BRCA: breast invasive carcinoma; COAD: colon adenocarcinoma; ESCA: esophageal carcinoma; HNSC: head and neck squamous cell carcinoma; KICH: kidney chromophobe; KIRC: kidney renal clear cell carcinoma; KIRP: kidney renal papillary cell carcinoma; LIHC: liver hepatocellular carcinoma; LUAD: lung adenocarcinoma; LUSC: lung squamous cell carcinoma; PRAD: prostate adenocarcinoma; STAD: stomach adenocarcinoma

**Figure 2:**
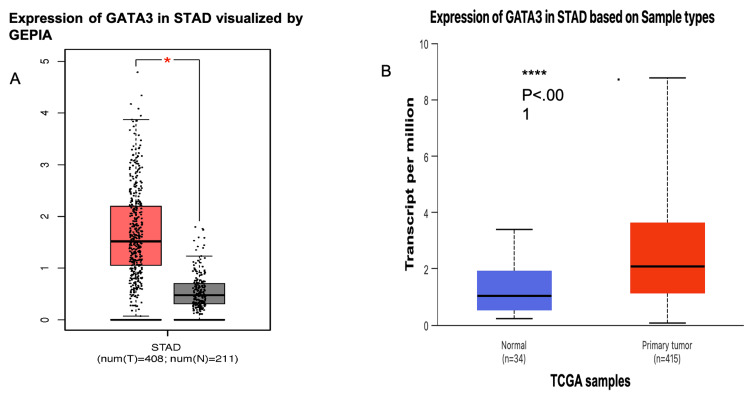
Expression profiling of GATA3 visualized by (A) GEPIA and (B) UALCAN In 2A, the red column represents the tumor tissues and the grey columns represent the normal tissues, while the stars explain the significance between the tumor and normal samples; P< 0.001 STAD: stomach adenocarcinoma; UALCAN: University of Alabama at Birmingham Cancer Data Analysis Portal [17]; N: normal; T: tumor; Num: number; GEPIA: Gene Expression Profiling Interactive Analysis database [16]; TCGA: The Cancer Genome Atlas [15]

GATA3 expression profile across TCGA and GTEx data analyzed by TIMER showed upregulation in five tumor types, including bladder urothelial carcinoma (BLCA), breast invasive carcinoma (BRCA), head and neck carcinoma (HNSC), esophageal carcinoma (ESCA), and STAD. Contrarily, GATA3 expression was downregulated in kidney chromophobe (KICH), kidney renal clear cell carcinoma (KIRC), kidney renal papillary cell carcinoma (KIRP), liver hepatocellular carcinoma (LIHC), lung adenocarcinoma (LUAD), lung squamous cell carcinoma (LUSC), prostate adenocarcinoma (PRAD), and colon adenocarcinoma (COAD), as shown in Figure 1. Therefore, investigation on GEPIA (Figure 2A) and UALCAN (Figure 2B) showed upregulation of GATA3 with significant differential expression. All three databases showed a highly significant differential expression across STAD since it was found highly upregulated (P < 0.001).

Analysis of the GATA3 expression across different clinicopathological parameters

We investigated GATA3 expression in stomach cancer across clinicopathological parameters, including individual cancer stages (I-IV), sex groups, ethnic differences, especially among Caucasian, African American, and Asian populations, and age comparisons between patients aged 21 to 100 years.

**Figure 3:**
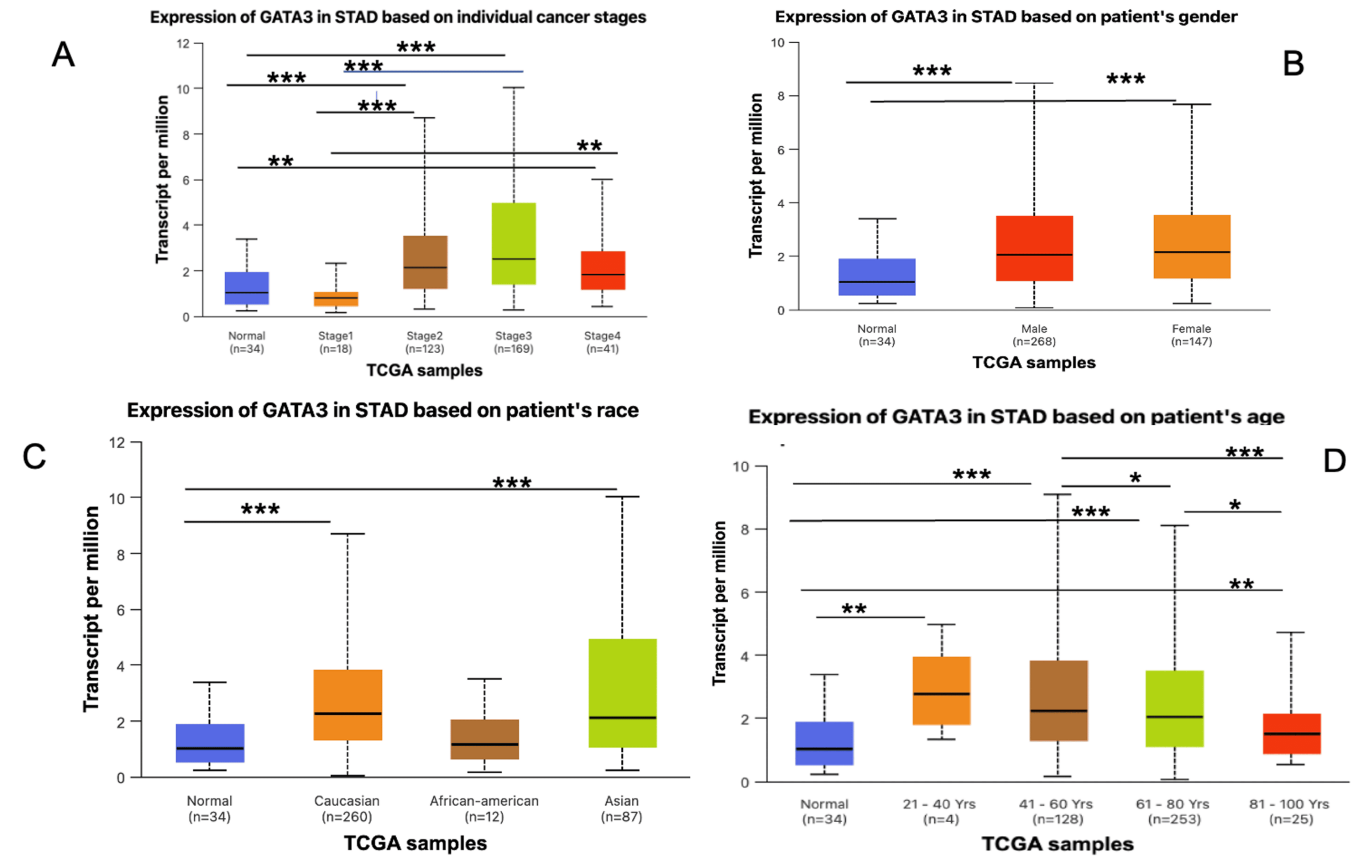
Correlation between the GATA3 expression and clinicopathological parameters (stage, gender, race, age) in STAD using UALCAN data (A) Expression of GATA3 based on individual cancer stage; (B) Expression of GATA3 based on gender including male and female; (C) Expression of GATA3 based on patient race; (D) Expression of GATA3 based on patient age Significant upregulation observed in stage II-III, Caucasian population, and age group 41-80 years (***P<0.001) *p < 0.05, **p < 0.01, ***p < 0.001 was assumed to be significant. UALCAN: University of Alabama at Birmingham Cancer Data Analysis Portal [17]

The outcomes revealed that the expression of GATA3 was significantly elevated in stages II and III compared to normal tissue, while lower expression was observed in stages I and IV. Between stages, GATA3 was highly expressed in stages II and III compared to stage I, as shown in Figure 3A. GATA3 expression was extremely significantly upregulated in both male and female groups (P-value <0.001), as illustrated in Figure 3B. Regarding racial groups, GATA3 expression was most significantly upregulated in the Caucasian group (P < 0.001), followed by the Asian group, as shown in Figure 3C. Furthermore, GATA3 expression in STAD was significantly upregulated in the age groups of 41-60 and 61-80 years compared to normal tissue (P< 0.001), as displayed in Figure 3D. Besides, GATA3 expression was significantly upregulated in the age groups of 41-60 and 81-100 years, whereas slightly significant differences were observed in the age groups of 61-80, 41-60, and 81-100 years. These results emphasize stage-specific, population-based, and age-related variations in GATA3 expression in gastric carcinoma.

Immunohistochemical analysis 

Immunohistochemical (IHC) data were obtained from the HPA using the validated anti-GATA3 antibody CAB016217, ensuring reproducibility across samples. STAD tissue samples were formalin-fixed and paraffin-embedded (FFPE), and GATA3 protein expression was visualized using 3,3′-diaminobenzidine (DAB) staining. Staining intensity was semi-quantitatively assessed as negative, weak, moderate, or strong, and the fraction of stained tumor cells was recorded, focusing specifically on tumor epithelial cells.

**Figure 4:**
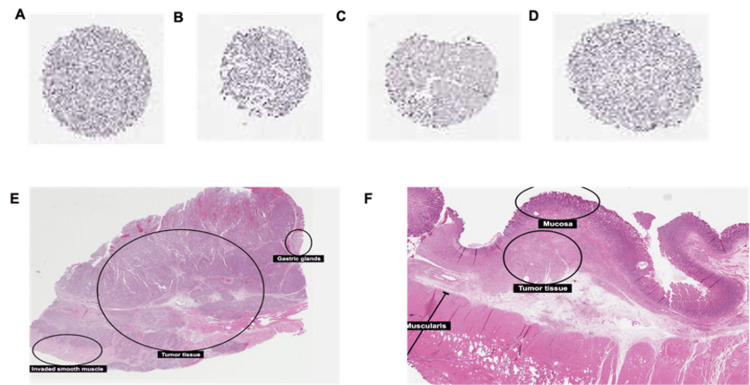
Immunohistochemistry of STAD tissue show moderate to strong cytoplasmic GATA3 staining (A-F),consistent across histological subtypes (A) Male, age 69, Stomach, upper (T-62350) Adenocarcinoma; Stomach cancer CAB016217 (https://www.proteinatlas.org/ENSG00000107485-GATA3/cancer/stomach+cancer#img); (B) Male, age 80, Stomach, upper (T-62350) Adenocarcinoma; Stomach cancer CAB016217 (https://www.proteinatlas.org/ENSG00000107485-GATA3/cancer/stomach+cancer#img); (C) Male, age 47, Stomach, (T-63000) Adenocarcinoma; Stomach cancer CAB016217 (https://www.proteinatlas.org/ENSG00000107485-GATA3/cancer/stomach+cancer#img); (D) Female, age 81, Stomach, upper (T-62350) Adenocarcinoma, NOS (M-81403) (https://www.proteinatlas.org/ENSG00000107485-GATA3/cancer/stomach+cancer#img); (E) Male, 60 years, moderately differentiated adenocarcinoma, intestinal type (https://www.proteinatlas.org/learn/dictionary/cancer/stomach+cancer#Stomach-cancer-1,-adenocarcinoma); (F) Male, 52 years, poorly differentiated adenocarcinoma, diffuse type (https://www.proteinatlas.org/learn/dictionary/cancer/stomach+cancer#Stomach-cancer-2,-adenocarcinoma) Image Source: Human Protein Atlas Version: 24.0 [18]; under CC BY-SA 4.0, Attribution-ShareAlike 4.0 International. URLs that link directly to each image are given in the above descriptions according to the requirements of HPA. STAD: stomach adenocarcinoma

All six STAD samples analyzed demonstrated moderate to strong cytoplasmic GATA3 expression, regardless of patient age (47-81 years), sex, or histological subtype, including both intestinal-type (moderately differentiated) and diffuse-type (poorly differentiated) adenocarcinomas (Figure 4). No negative or weak staining was observed, supporting the reproducibility and reliability of the HPA data for diagnostic assessment. Notably, GATA3 localization was predominantly cytoplasmic, which may indicate a non-canonical role in tumor biology.

Importantly, these IHC findings are consistent with our transcriptomic analyses from TIMER, GEPIA, UALCAN, and GEO datasets, which revealed significant upregulation of GATA3 mRNA in STAD tissues compared to normal tissues (p < 0.001). The concordance between mRNA and protein expression strengthens the evidence that GATA3 may serve as a robust diagnostic biomarker for STAD and highlights its potential involvement in tumor differentiation and immune microenvironment modulation.

Prognostic evaluation of GATA3 in STAD

**Figure 5:**
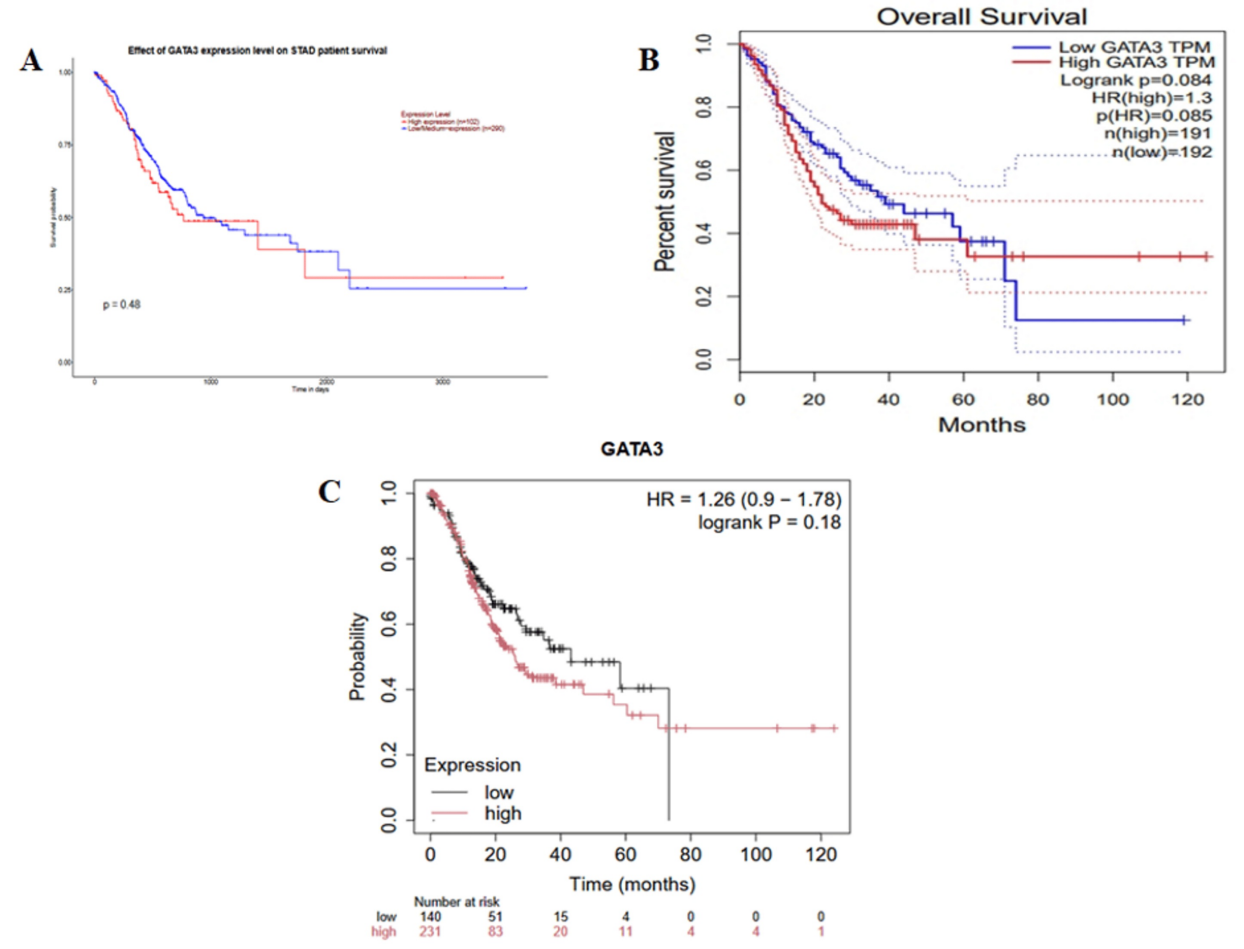
Association of overall survival with GATA3 expression according to UALCAN, GEPIA, and KM Plotter (A) UALCAN overall survival analysis comparing STAD patient with high GATA3 expression (n=102)versus low expression (n=290); (B) GEPIA overall survival analysis stratified by median GATA3 expression (high=191; low=192). HR=1.3; (C) KM Plotter analysis survival in high (n=231) versus low (n=140) GATA3 expressers. HR=1.26; The red line represents high gene expression, and the blue line represents low gene expression. no significant survival association (P>0.05) UALCAN : University of Alabama at Birmingham Cancer Data analysis portal [17]; GEPIA: Gene Expression Profiling Interactive Analysis database [16]; KM Plotter: Kaplan-Meier plotter; HR: hazard ratio

The findings from GEPIA, UALCAN, and KM plotter datasets were extracted in the form of a KM curve, as it explains the correlation of GATA3 expression over time. Figure 5A shows UALCAN analysis of overall survival in STAD patients. The cohort included 102 patients with high GATA3 expression and 290 patients with low to medium expression. The difference between the groups was not statistically significant (p > 0.05). Patients with high GATA3 expression tended to have poorer prognosis compared with those with low or medium expression. Figure 5B represents the OS rate from GEPIA, emphasizing no significance associated with gene expression and prognosis (p>0.05). This confirms poor prognosis in high GATA3 expression, which is represented by a sample size of 191, but also a good prognosis in low expression of 192 samples, with a lower HR of 1.3. Therefore, the KM plotter in Figure 5C comes as a confirming bioinformatic finding that the prognostic potential usage of GATA3 is not valid with p>0.05. However, the prognosis pattern is seen as poor prognosis in high gene expression (231 samples) and good prognosis in low expression (140 samples with an HR of 1.26. These findings suggest that high GATA3 expression may influence prognosis; however, since the association was not statistically significant, GATA3 cannot be considered a reliable prognostic biomarker in STAD.

Gene-immune cell infiltration analysis

**Figure 6:**
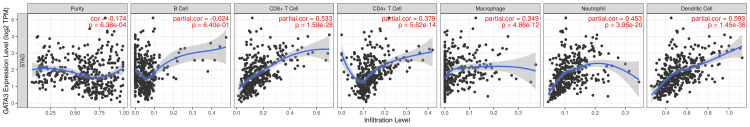
GATA3 immune-cell infiltration using the TIMER database tumor purity - Correlation of GATA3 expression with immune cells infiltration levels; significant positive correlation in CD8+ T cells (partial cor = 0.533, p<0.0001), CD4+ T cells (parial cor = 0.379, p<0.0001), macrophages (parial cor = 0.349, p<0.0001), neutrophils (parial cor = 0.453 p<0.0001), and dendritic cells (parial cor = 0.593, p<0.0001), no significant correlation was absorved with (B cell ,P>0.05) in STAD as assessed by the TIMER database. Partial cor: partial Spearman’s correlation coefficient, adjusted for tumor purity; log2 TPM: logarithm of 2 transcripts per million; STAD: stomach adenocarcinoma; TIMER: Tumor Immune Microenvironment Estimation and Resource [14]

Tumor microenvironment mostly integrates intracellular, intercellular, and extracellular components, particularly immune cells, including B cells, CD8+ T cells, CD4+ T cells, macrophages, neutrophils, and dendritic cells, to understand the full function of sub-cellular or cellular mechanisms, and that results in better mentoring the therapeutic process. Therefore, the gene-cell immune infiltrations across STAD showed the possible correlation between GATA3 expression and immune cells by examining in silico purity abundance in the form of positive or negative correlation, where GATA3 expression is significantly negatively correlated with tumor purity (p<0.001). GATA3 expression showed substantial strong positive correlation with several immune cell types, which was revealed by a highly significant p<0.0001 noticeably in CD8+ T cells (partial Spearman’s correlation coefficient, adjusted for tumor purity (partial cor) = 0.533), CD4+ T cells (partial cor = 0.379), macrophages (partial cor = 0.349), neutrophils (partial cor = 0.453), and dendritic cells (partial cor = 0.593). However, it showed negative insignificance with B cells (p>0.05, partial cor = 0.024), as shown in Figure 6. In conclusion, STAD demonstrated a positive association with specific immune cells, while GATA3 expression showed a negative correlation with tumor purity. These findings suggest that GATA3 expression impacts immune responses by modulating immune cell infiltration within the tumor microenvironment, potentially offering diagnostic and therapeutic implications in relation to understanding and mentoring any sort of immunotherapy

DNA methylation analysis

**Figure 7:**
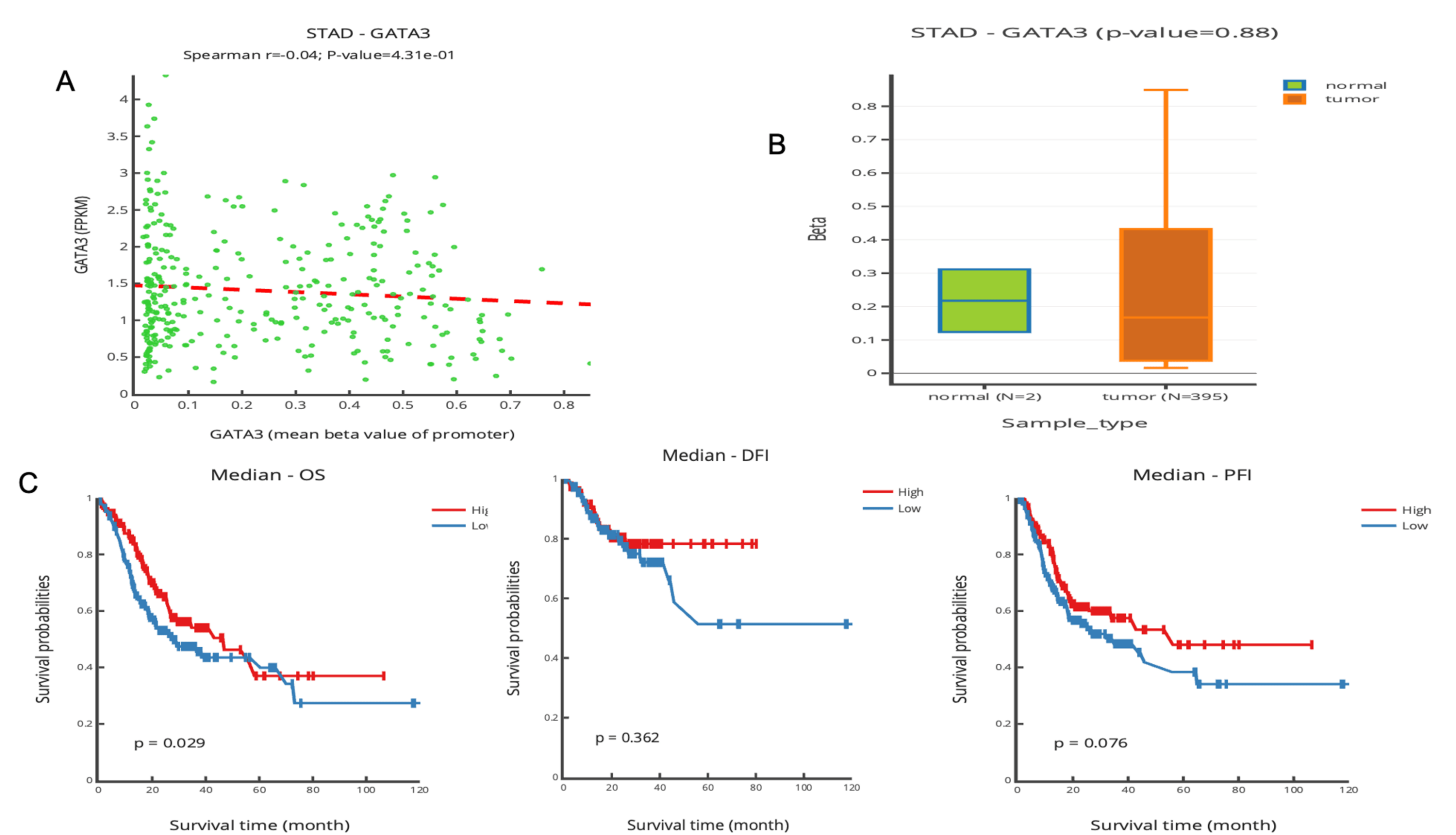
Epigenetic regulation of GATA3 through promoter methylation in STAD (A) DNMIVD heatmap shows hypermethylation in STAD (p<0.001); (B) the DNA methylation level of the GATA3 promoter region between normal tissue and STAD tissue spearman correlation (p< 0.05); (C) Kaplan-Meier survival curve of DNA methylation and prognosis  of the GATA3 gene in STAD (p<0.029) DNMIVD: DNA Methylation Interactive Visualization Database [19]; STAD: stomach adenocarcinoma

Studying the potential affinity between DNA methylation and STAD prognosis using datasets from TCGA, GEO, and DNMIVD showed a hypermethylation of the DNA promoter in correlation to GATA3 expression (p<0.001) (Figure 7A). Meanwhile, Spearman's curve revealed a significant negative affinity (r= -0.04, p<0.05) between the value of the promoter and gene expression (Figure 7B). The outcomes revealed that there are other mechanisms for increasing the expression level of GATA3, and further experiments are required to uncover them. Correspondingly, Figure 7C explored the association between gene methylation and prognosis in STAD in terms of OS rate, which is remarkably observed as a significantly good prognosis as GATA3 expression is low (p<0.0001).

Genomic analysis of gene alterations

**Figure 8:**
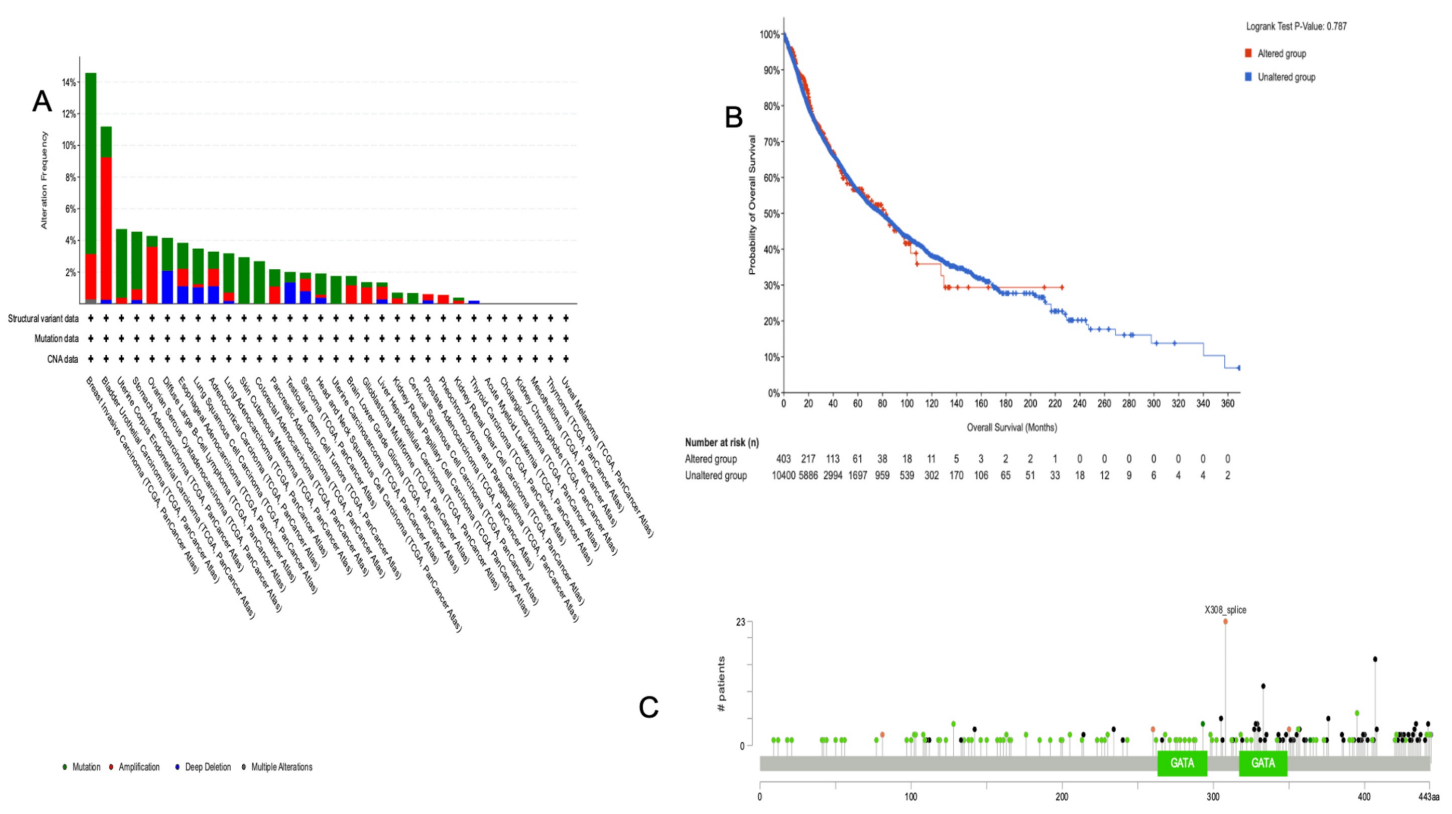
Genomic alterations of GATA3 across stomach adenocarcinoma using the cBioPortal platform (A) Distribution of genetic alteration frequencies of GATA3 across 32 cancer type from TCGA PanCan Atlas [24] (n=10967 samples); STAD shows 4.55% alteration rate (mutation 3.64%,amplifications 0.68%,deep deletions 0.23%). (B) Survival analysis comparing patients with STAD and GATA3 alteration (p>0.05). (C) Lollipop plot highlighting mutation site in GATA3. cBioPortal: Cancer Bioinformatic Portal for Cancer Genomic [21]; STAD: stomach adenocarcinoma; TCGA: The Cancer Genome Atlas [15]

The genomic analysis in cBioPortal, which investigates genetic alterations in GATA3 expression across different types of tumors, revealed that the frequency of GATA3 mutation was 4% in 10,967 samples from 32 studies. As plotted in Figure 8A, the distribution of the GATA3 variant across various tumor types since the elevated alteration frequencies of GATA3 are most prevalent in BRCA, with an incidence rate of 14.58% in 1084 samples, followed by BLCA (11.19%), UCS (4.73%), and STAD (4.55%). However, in BRCA, the *GATA3* gene is present in 1084 cases with various alteration frequencies, including predominantly mutation in 11.44% (124 cases), amplification in 2.86% (31 cases), and other unlisted multiple alterations in 0.28% (three cases). In contrast, in STAD, the gene was altered in 440 cases, with alteration frequencies of mutation in 3.64% (16 cases), amplification in 0.68% (three cases), and deep deletion in 0.23% (one case).

In addition, these findings represent deep observations suggesting that GATA3 alterations play an essential role in the pathogenesis of characteristic neoplasms, particularly in demonstrating high alteration frequencies. The bioinformatic genomic analysis shows a potential method for therapeutic intervention for these malignancies, particularly where extracted data from the KM curve in Figure 8B compares the OS between patients with GATA3 alterations and those without alterations. Consequently, the number of patients at risk was presented at specified intervals, exhibiting that the unmodified group remained larger and consistent throughout the study period, with no significance observed between the two groups, including the unaltered and altered.

Nonetheless, the lollipop plot in Figure 8C explains the significant identified mutating amino acids in accordance with the GATA3 single cell sequence of the analyzed data, where each point represents a mutation with different colors showing the mutation type or specific sites with a higher frequency of mutations. Therefore, the key GATA3 functional domains are highlighted in green, demonstrating regions where mutations are focused, particularly missense mutations. In conclusion, this comprehensive genomic gene in pan-cancer analysis revealed that GATA3 alterations are prevalent in several cancer types with varying frequencies, of which survival analysis suggests a potential good correlation with unaltered GATA3 alterations. This suggests the need for further investigation to determine its statistical significance in carcinogenesis.

Open-data lab-based validation

**Figure 9:**
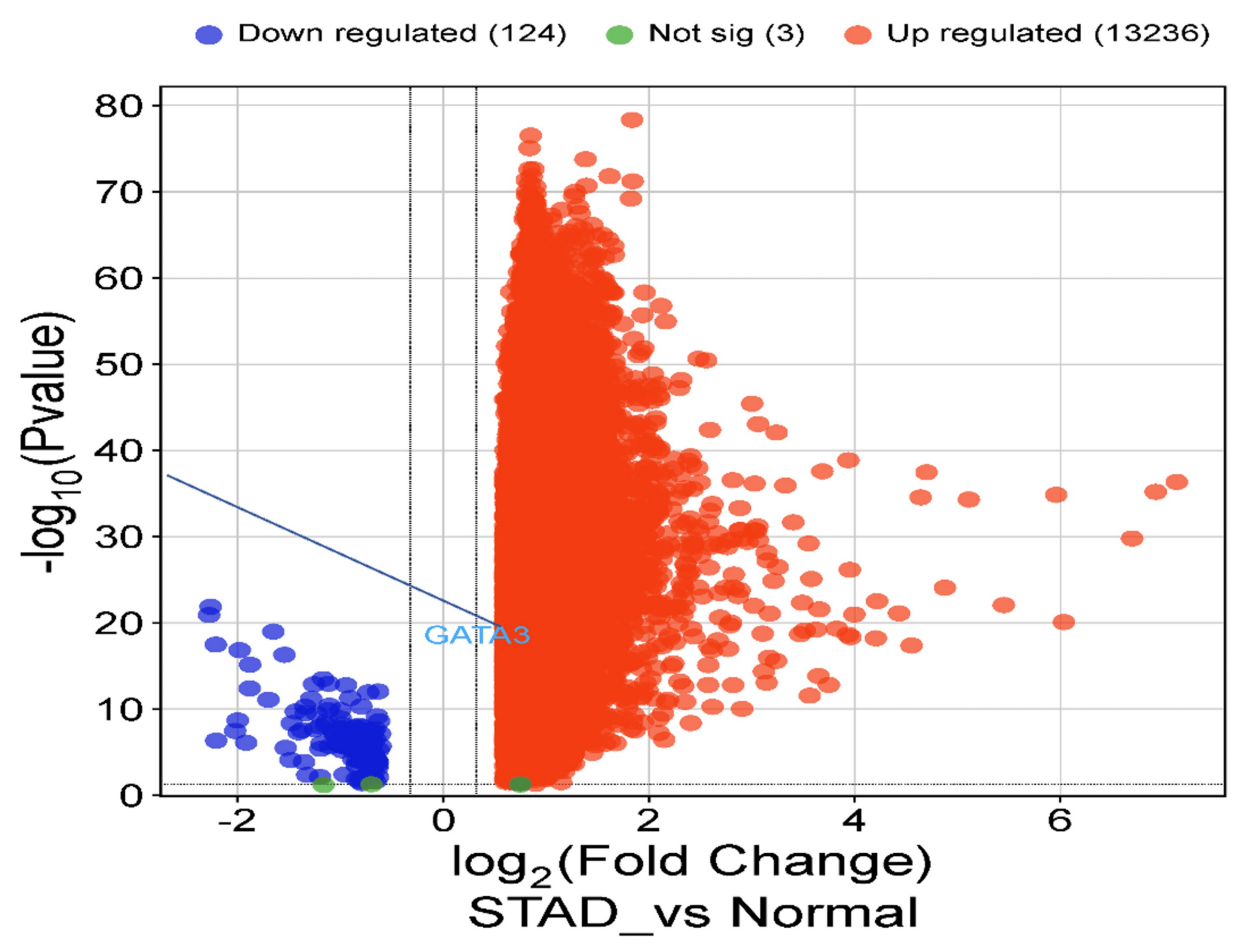
Volcano plot of differential gene expression in STAD including GATA3 gene Volcano plot illustrating differential gene expression, including GATA3 in STAD. Upregulated genes are shown as red dots, and downregulated genes are shown as blue dots (|Log2FC| > 0.6, adj p-value < 0.05). GATA3: GATA binding protein 3; STAD: stomach adenocarcinoma.

Given that this analysis presents great insights regarding the potential usage of GATA3 as an essential diagnostic biomarker and therapeutic mechanism, a validation is needed. Accordingly, the outcomes of utilizing dataset GSE29272 based on the statistical parameters of adj-p<0.05 and log2FC>0.6, the upregulation of GATA3 in STAD is plotted in a volcano plot using the SRplot web-based tool (Figure 9). The validation analysis revealed 13,236 upregulated genes, including GATA3, and 124 downregulated genes. 

## Discussion

This study comprehensively investigated the expression, diagnostic potential, prognostic significance, immune interactions, methylation status, and genetic alterations of GATA3 in STAD using multiple public bioinformatics platforms. Our findings highlight the potential role of GATA3 as a diagnostic biomarker, while also revealing its complex regulatory mechanisms and interactions within the tumor immune microenvironment.

GATA3, a transcription factor involved in epithelial cell differentiation, development, and tumor biology, has previously demonstrated diagnostic value in breast and urothelial carcinomas [25]. Our analyses revealed significant upregulation of GATA3 in STAD tissues compared to normal tissues across TIMER, GEPIA, and UALCAN. This upregulation was further validated using the independent GEO dataset GSE29272, where GATA3 expression was significantly elevated (adjusted p < 0.05, log2FC > 0.6). The consistent upregulation across multiple datasets suggests that GATA3 may serve as a diagnostic marker in STAD.

Although GATA3 expression was elevated, our survival analysis via GEPIA, UALCAN, and KM Plotter did not show a statistically significant association between GATA3 expression levels and overall survival in STAD patients. While our study indicates that GATA3 overexpression in STAD correlates with immune cell infiltration, it does not associate with poor prognosis, suggesting a role in maintaining tumor differentiation rather than promoting aggressive progression. This observation aligns with findings in other cancers, where GATA3 expression is often linked to less aggressive tumor phenotypes. For instance, in breast cancer, GATA3 is associated with luminal subtypes and favorable clinical outcomes, contrasting with the functions of other GATA family members [26].

In contrast, other GATA family members exhibit distinct and sometimes opposing roles in cancer progression. GATA4 and GATA6, for example, are implicated in promoting tumorigenesis in various cancers, including gastric cancer. Aberrant expression of GATA4 and GATA6 contributes to oncogenesis due to their roles as pioneer transcription factors, which remodel chromatin and facilitate binding of additional transcription factors and cofactors, ultimately favoring tumor progression [27]. The prognostic significance of GATA family members varies across cancer types; for instance, in ovarian cancer, high expression of GATA1, GATA2, and GATA4 correlates with better OS, whereas increased expression of GATA3 and GATA6 is associated with poorer outcomes [28]. These contrasting roles underscore the complexity of GATA transcription factors in cancer biology and suggest that each member contributes uniquely to tumor behavior depending on the tissue context.

Immune infiltration analysis revealed that GATA3 expression positively correlates with several immune cell types, including CD8+ T cells, CD4+ T cells, macrophages, dendritic cells, and neutrophils, highlighting its potential role in modulating the tumor immune microenvironment and relevance to immunotherapy in STAD. However, further experimental studies are needed to clarify the precise mechanisms by which GATA3 influences immune cell activation and tumor-immune interactions.

Interestingly, promoter methylation analysis showed elevated methylation levels in STAD tissues despite increased GATA3 expression. This apparent inconsistency may reflect complex regulatory mechanisms, such as post-transcriptional modifications or region-specific promoter activity, which can override the inhibitory effects of methylation [29]. Such findings underscore the importance of integrated multi-omics approaches to fully understand gene regulation in cancer.

Genomic analysis using cBioPortal indicated that GATA3 mutations are relatively rare in STAD (4.55%), and survival analyses revealed no significant impact of these mutations on patient outcomes. Collectively, these data suggest that GATA3 dysregulation in STAD occurs primarily through transcriptional or epigenetic mechanisms rather than genetic alterations.

Overall, our findings indicate that GATA3 is consistently upregulated in STAD and may serve as a valuable diagnostic biomarker. Its association with immune cell infiltration highlights potential immunomodulatory functions, while its lack of correlation with poor prognosis supports the hypothesis that GATA3 maintains tumor differentiation rather than driving aggressive progression. However, the different roles of other GATA family members emphasize the need for a precise understanding of these transcription factors in oncogenesis. Future studies should investigate the differential functions of GATA family members across cancers to elucidate their diagnostic, prognostic, and therapeutic potential.

Limitations

The biological role of GATA3 in STAD progression, metastasis, or therapy response remains unexplored in vitro or in vivo. Variability among datasets regarding sample preparation, sequencing platforms, and clinical annotations may influence the results. The treatment modalities, response to immunotherapy were not integrated due to limited data availability. Clinical validation of functional overexpression is essential to confirm the bioinformatics findings and understand GATA3’s biological function in STAD. Also, we did not investigate how GATA3 expression affects tumor-immune interactions, and whether it could serve as a predictive biomarker for immunotherapy response.

## Conclusions

This study provides a comprehensive bioinformatics analysis of GATA3 in STAD. We demonstrated that GATA3 is significantly upregulated in STAD tissues, highlighting its potential properties as a diagnostic biomarker. The gene’s expression correlates with various immune cell infiltrates, revealing its immunomodulatory role; prognostic significance remains limited, as no statistically significant association with OS was observed. The study also revealed that GATA3 promoter methylation levels are elevated in STAD. Additionally, the low frequency of genetic alterations and effect on survival outcomes further support the hypothesis that GATA3 dysregulation in STAD may occur primarily through transcriptional or epigenetic mechanisms. The findings underline the diagnostic relevance of GATA3 in STAD and highlight the need for further research to clarify its functional role in tumor biology and the immune microenvironment.
